# Clinical Outcomes Following Treatment for COVID-19 With Nirmatrelvir/Ritonavir and Molnupiravir Among Patients Living in Nursing Homes

**DOI:** 10.1001/jamanetworkopen.2023.10887

**Published:** 2023-04-27

**Authors:** Bosco Hon-Ming Ma, Terry Cheuk-Fung Yip, Grace Chung-Yan Lui, Mandy Sze-Man Lai, Elsie Hui, Vincent Wai-Sun Wong, Yee-Kit Tse, Henry Lik-Yuen Chan, David Shu-Cheong Hui, Timothy Chi-Yui Kwok, Grace Lai-Hung Wong

**Affiliations:** 1 Department of Medicine and Therapeutics, The Chinese University of Hong Kong, Hong Kong; 2 Medical Data Analytics Centre, The Chinese University of Hong Kong, Hong Kong; 3 Institute of Digestive Disease, The Chinese University of Hong Kong, Hong Kong; 4 Stanley Ho Centre for Emerging Infectious Diseases, Jockey Club School of Public Health and Primary Care, The Chinese University of Hong Kong, Hong Kong; 5 Faculty of Medicine, The Chinese University of Hong Kong, Hong Kong; 6 Department of Internal Medicine, Union Hospital, Hong Kong

## Abstract

**Importance:**

Older patients living in nursing homes are at very high risk of mortality after getting COVID-19.

**Objective:**

To evaluate outcomes following oral antiviral treatment for COVID-19 among nonhospitalized older patients living in nursing homes.

**Design, Setting, and Participants:**

This is a territory-wide, retrospective cohort study conducted between February 16 and March 31, 2022, with the last follow-up date on April 25, 2022. Participants were patients with COVID-19 living in nursing homes in Hong Kong. Data analysis was performed from May to June 2022.

**Exposures:**

Molnupiravir, nirmatrelvir/ritonavir, or no oral antiviral treatment.

**Main Outcomes and Measures:**

The primary outcome was hospitalization for COVID-19, and the secondary outcome was risk of inpatient disease progression (ie, admission to intensive care unit, use of invasive mechanical ventilation, and/or death).

**Results:**

Of 14 617 patients (mean [SD] age, 84.8 [10.2] years; 8222 women [56.2%]), 8939 (61.2%) did not use oral antivirals, 5195 (35.5%) used molnupiravir, and 483 (3.3%) used nirmatrelvir/ritonavir. Compared with patients who did not use oral antivirals, those who used molnupiravir and nirmatrelvir/ritonavir were more likely to be female and less likely to have comorbid illnesses and hospitalization in the past year. At a median (IQR) follow-up of 30 (30-30) days, 6223 patients (42.6%) were hospitalized and 2307 patients (15.8%) experienced inpatient disease progression. After propensity score weighting, both molnupiravir and nirmatrelvir/ritonavir were associated with a reduced risk of hospitalization (molnupiravir, weighted hazard ratio [wHR], 0.46; 95% CI, 0.37-0.57; *P* < .001; nirmatrelvir/ritonavir, wHR, 0.46; 95% CI, 0.32-0.65; *P* < .001) and inpatient disease progression (molnupiravir, wHR, 0.35; 95% CI, 0.23-0.51; *P* < .001; nirmatrelvir/ritonavir, wHR, 0.17; 95% CI, 0.06-0.44; *P* < .001). Nirmatrelvir/ritonavir was comparable to molnupiravir in achieving better clinical outcomes (hospitalization, wHR, 1.00; 95% CI, 0.75-1.33; *P* = .99; inpatient disease progression, wHR, 0.49; 95% CI, 0.20-1.20; *P* = .12).

**Conclusions and Relevance:**

In this retrospective cohort study, the use of oral antivirals to treat COVID-19 was associated with a reduced risk of hospitalization and inpatient disease progression among patients living in nursing homes. The findings of this study of nursing home residents could be reasonably extrapolated to other frail older patients living in the community.

## Key Points

**Question:**

What is the association between the use of 2 oral antivirals, molnupiravir and nirmatrelvir/ritonavir, and the risk of hospitalization and inpatient disease progression among patients with COVID-19 living in nursing homes?

**Findings:**

In this cohort study of 14 617 patients, both molnupiravir and nirmatrelvir/ritonavir treatment were associated with a lower risk of hospitalization and a lower risk of inpatient disease progression.

**Meaning:**

These findings suggest that the use of oral antivirals to treat COVID-19 may benefit older patients living in nursing homes by decreasing risks of hospitalization and inpatient disease progression.

## Teaser

This cohort study evaluates outcomes following oral antiviral treatment for COVID-19 among nonhospitalized older patients living in nursing homes.

## Introduction

The COVID-19 pandemic poses an unprecedented challenge to health care systems all over the world, especially for curbing outbreaks in nursing homes, which are associated with very high mortality.^1,2,3,4^ The emergence of 2 novel oral antivirals, molnupiravir and nirmatrelvir/ritonavir, is groundbreaking in that treatment is available for nonhospitalized patients who are at risk of deterioration. It is hoped that the timely commencement of oral antiviral therapy, within 5 days of symptom onset, can halt and/or mitigate the risk of disease progression (ie, hospitalization, use of invasive mechanical ventilation [IMV], admission to intensive care unit [ICU], or death). The efficacy is supported by the results of 2 randomized clinical trials (RCTs)^5,6^ for nonhospitalized patients published in early 2022. In the MOVe-OUT study,^5^ molnupiravir reduced the risk of hospitalization or death compared with placebo (6.8% vs 9.7%) with relative risk reduction of 30% and number needed-to-treat of 34. In the EPIC-HR study,^6^ nirmatrelvir/ritonavir lowered the risk of hospitalization or death compared with placebo (0.72% vs 6.53%) with relative risk reduction of 89% and number needed to treat of 17. Clinicians should note that the study patients were relatively young. Their median age was 43 years in the MOVe-OUT study^5^ and 46 years in the EPIC-HR study.^6^

There is a paucity of data on the clinical effectiveness of these oral antivirals, especially among older patients with multiple comorbidities and disability. In Hong Kong, molnupiravir and nirmatrelvir/ritonavir have been available since March 2, 2022, and March 11, 2022, respectively. Our group has previously shown that the use of nirmatrelvir/ritonavir, but not molnupiravir, was associated with a reduced risk of hospitalization in nonhospitalized, community-dwelling patients with COVID-19 whose mean age was 49 years.^7^ It is uncertain whether the oral antivirals are effective in reducing the risk of hospitalization or inpatient disease progression among older patients. To supplement our previous work,^7^ the present study aimed to evaluate the clinical effectiveness of 2 oral antivirals for COVID-19 in nonhospitalized older patients living in nursing homes who were more frail than their community-dwelling counterparts.

## Methods

### Study Design

A territory-wide retrospective cohort study was conducted using the electronic database Clinical Data Analysis and Reporting System (CDARS) of the Hospital Authority, which manages all public hospitals of Hong Kong.^8^ CDARS captures patients’ demographic data, diagnoses, procedures, surgery, medications, laboratory investigations, radiology, clinical appointments, and death from all public clinics and hospitals, representing inpatient and outpatient care for approximately 80% of the 7.4 million local population. The *International Classification of Diseases, Ninth Revision, Clinical Modification* coding system was adopted in CDARS. CDARS can identify nearly all medical conditions.^9^ Territory-wide studies on COVID-19 using CDARS have been published elsewhere.^9,10^ Data were deidentified in CDARS, so informed consent was not sought in accordance with our institution’s policy. This study was reported following the Strengthening the Reporting of Observational Studies in Epidemiology (STROBE) reporting guideline. This study was approved by the Joint Chinese University of Hong Kong New Territories East Cluster Clinical Research Ethics Committee.

### Patients

This study analyzed older nursing home patients who received care for COVID-19 from community geriatric assessment teams (CGATs) in Hong Kong between February 16 and March 31, 2022. The Hospital Authority has 15 CGATs in 7 geographic clusters (Hong Kong East, Hong Kong West, Kowloon Central, Kowloon East, Kowloon West, New Territories East, and New Territories West), covering nearly all nursing homes in Hong Kong. Each CGAT is composed of geriatricians and nurses who collaborate closely with nursing homes. Medical consultations were delivered either in nursing homes or via telemedicine care. All patients had COVID-19 confirmed by polymerase chain reaction test and/or diagnosis code (519.8:8). Molnupiravir or nirmatrelvir/ritonavir was prescribed for those at high risk of deterioration and within 5 days of symptom onset after they were available on March 2, 2022, and March 11, 2022, respectively (eTable 1 in Supplement 1). The criteria for high risk of deterioration varied with time because of the availability of the 2 antivirals in Hong Kong. Patients were excluded if they were hospitalized on the day of first consultation or prescribed both molnupiravir and nirmatrelvir/ritonavir. Patients were followed up until the occurrence of any of the following end points, whichever came first: clinical end point of interest, death, date of data retrieval (April 25, 2022), or up to 30 days of follow-up.

The baseline date was defined as the first day of CGAT consultation. Data including demographic characteristics, hospitalization, diagnoses, procedures, blood tests (hemoglobin A_1c_, fasting glucose, complete blood picture, liver biochemistry, and kidney function), COVID-19 polymerase chain reaction test, and use of antivirals (molnupiravir and/or nirmatrelvir/ritonavir) were collected.

### Definitions

The primary end point was the time to first hospitalization. The secondary end point was a composite outcome of inpatient disease progression: ICU admission, use of IMV, and/or death. Hospitalization was defined as staying in hospital for more than 1 day. Date and cause of death were ascertained using data from CDARS and the Hong Kong Death Registry. IMV use was defined by *International Classification of Diseases, Ninth Revision, Clinical Modification* procedure codes (96.04-96.05, 96.7). Definition of comorbidities are described in the eAppendix and eTable 2 in Supplement 1.

### Statistical Analysis

Data analysis was performed from May to June 2022. Data were analyzed using R statistical software version 4.1.3 (R Project for Statistical Computing).^11^ Continuous variables were expressed as mean (SD) or median (IQR [25th-75th percentile]), whereas categorical variables were presented as frequency (percentage). Qualitative and quantitative differences between groups were compared by χ^2^ test or Fisher exact test for categorical parameters, and 1-way analysis of variance or Kruskal-Wallis test for continuous parameters.

Propensity score (PS) of oral antiviral nonusers, molnupiravir users, and nirmatrelvir/ritonavir users was estimated by multinomial logistic regression on 18 clinical characteristics, including age, sex, baseline date, cardiovascular disease, digestive disease, diabetes, malignant tumor, nervous system disease, respiratory disease, kidney disease, hemoglobin, white blood cell, platelets, creatinine, alanine aminotransferase, albumin, total bilirubin, and number of hospitalizations in the past years; the 7 geographic clusters of nursing home was included as a random intercept.^12^ Average treatment effect weighting was used for those treated with nirmatrelvir/ritonavir. The balance of clinical characteristics between groups was assessed by absolute standardized mean difference, with a value of less than 0.1 indicating a good balance.^13^ Clinical characteristics with absolute standardized mean difference of 0.1 or higher after PS weighting were adjusted in the weighted Cox model for double robustness. Use of molnupiravir and nirmatrelvir/ritonavir was modeled as a time-dependent covariate in the weighted Cox model. Details on multiple imputation and Cox model and 5-day landmark analysis are described in the eAppendix in Supplement 1. All statistical tests were 2-sided. Statistical significance was taken as *P* < .05.

## Results

### Demographic Characteristics

After excluding 391 patients because they were hospitalized on the day of first consultation and 13 patients who were prescribed both antivirals (eFigure 1 in Supplement 1), a total of 14 617 patients (mean [SD] age, 84.8 [10.2] years; 8222 women [56.2%]) were analyzed. Table 1 describes the baseline clinical characteristics of the study patients.

**Table 1.** Clinical Characteristics of Patients With COVID-19 Who Received Care From Community Geriatric Assessment Team

Characteristics	Patients, No. (%)				*P* value^a^
	All (N = 14 617)	No oral antivirals (n = 8939)	Molnupiravir (n = 5195)	Nirmatrelvir/ritonavir (n = 483)	
Age, mean (SD), y	84.8 (10.2)	84.7 (10.4)	85.2 (9.8)	83.6 (10.1)	<.001
Sex					
Male	6395 (43.8)	4364 (48.8)	1849 (35.6)	182 (37.7)	<.001
Female	8222 (56.2)	4575 (51.2)	3346 (64.4)	301 (62.3)	
Comorbidities^b^					
Cardiovascular diseases	11 245 (76.9)	7027 (78.6)	3856 (74.2)	362 (74.9)	<.001
Hypertension	11 027 (75.4)	6852 (76.7)	3816 (73.5)	359 (74.3)	<.001
Ischemic heart disease	774 (5.3)	686 (7.7)	86 (1.7)	2 (0.4)	<.001
Cardiac dysrhythmias	1470 (10.1)	1180 (13.2)	277 (5.3)	13 (2.7)	<.001
Heart failure	847 (5.8)	724 (8.1)	120 (2.3)	3 (0.6)	<.001
Digestive diseases	1973 (13.5)	1583 (17.7)	361 (6.9)	29 (6.0)	<.001
Peptic ulcer	225 (1.5)	197 (2.2)	26 (0.5)	2 (0.4)	<.001
Chronic liver disease	882 (6.0)	626 (7.0)	235 (4.5)	21 (4.3)	<.001
Liver failure, cirrhosis, or cirrhotic complications	29 (0.2)	28 (0.3)	1 (0.02)	0	<.001
Biliary disease	372 (2.5)	332 (3.7)	40 (0.8)	0	<.001
Gastrointestinal hemorrhage	773 (5.3)	676 (7.6)	88 (1.7)	9 (1.9)	<.001
Diabetes	5309 (36.3)	3372 (37.7)	1786 (34.4)	151 (31.3)	<.001
Malignant tumors	518 (3.5)	449 (5.0)	60 (1.2)	9 (1.9)	<.001
Nervous system diseases	2334 (16.0)	2053 (23.0)	263 (5.1)	18 (3.7)	<.001
Cerebrovascular events	1687 (11.5)	1494 (16.7)	184 (3.5)	9 (1.9)	<.001
Other nervous system diseases	998 (6.8)	878 (9.8)	110 (2.1)	10 (2.1)	<.001
Respiratory diseases	1650 (11.3)	1392 (15.6)	240 (4.6)	18 (3.7)	<.001
Kidney diseases	1184 (8.1)	1060 (11.9)	119 (2.3)	5 (1.0)	<.001
HIV infection	13 (0.1)	9 (0.1)	2 (0.04)	2 (0.4)	.04
Laboratory parameters					
Hemoglobin, mean (SD), g/dL	11.6 (2.0)	11.5 (2.1)	11.7 (1.8)	12.1 (1.7)	<.001
Missing, %	4.6	2.0	8.3	12.4	
White blood cell count, mean (SD), cells/μL	8400 (4400)	8900 (4900)	7600 (3100)	7700 (2900)	<.001
Missing, %	4.6	2.0	8.3	12.4	
Platelet count, mean (SD), ×10^3^/μL	243.8 (102.2)	243.8 (108.2)	243.6 (91.3)	245.4 (91.2)	.92
Missing, %	4.6	2.0	8.3	12.4	
Creatinine, median (IQR), mg/dL	0.94 (0.72-1.33)	0.99 (0.75-1.49)	0.88 (0.71-1.17)	0.85 (0.70-1.01)	<.001
Missing, %	2.8	1.2	5.2	6.4	
Alanine aminotransferase, median (IQR), U/L	17 (12-26)	18 (12-28)	15 (11-22)	16 (11-23)	<.001
Missing (%)	4.2	1.7	7.9	11.4	
Albumin, mean (SD), g/dL	3.21 (0.60)	3.09 (0.59)	3.40 (0.56)	3.62 (0.56)	<.001
Missing (%)	4.4	1.7	8.3	11.8	
Total bilirubin, mean (SD), mg/dL	0.58 (0.53)	0.59 (0.60)	0.55 (0.36)	0.59 (0.34)	<.001
Missing (%)	4.5	1.8	8.5	12.2	
Time from the first date of inclusion, mean (SD), d^c^	17.1 (11.3)	13.3 (10.6)	22.8 (9.7)	27.1 (9.9)	<.001
Age- and sex-specified complete vaccination rate, mean (SD), %^d^	20.9 (12.8)	20.4 (12.8)	21.5 (12.6)	24.1 (13.7)	<.001
Hospitalizations in the past year, No. (%)					
0	10 626 (72.7)	5705 (63.8)	4507 (86.8)	414 (85.7)	<.001
1	2123 (14.5)	1518 (17.0)	548 (10.5)	57 (11.8)	
≥2	1868 (12.8)	1716 (19.2)	140 (2.7)	12 (2.5)	
Follow-up duration, median (IQR), d	30 (30-30)	30 (28-30)	30 (30-30)	30 (30-30)	<.001

SI conversion factors: To convert alanine aminotransferase to microkatals per liter, multiply by 0.0167; albumin to grams per liter, multiply by 10; bilirubin to micromoles per liter, multiply by 17.104; creatinine to micromoles per liter, multiply by 88.4; hemoglobin to grams per liter, multiply by 10; platelets to ×10^9 ^per liter, multiply by 1; white blood cells to ×10^9^ per liter, multiply by 0.001.

^a^ Qualitative and quantitative differences between subgroups were analyzed by χ^2^ or Fisher exact tests for categorical parameters and 1-way analysis of variance or Kruskal-Wallis test for continuous parameters, as appropriate.

^b^ All comorbidities were represented as binary parameters. The definition of comorbidities was stated in eTable 2 in Supplement 1.

^c^ Days from the first date of inclusion referred to the number of days from the first date of inclusion (ie, February 16, 2022).

^d^ Age- and sex-specified complete vaccination rate refers to the corresponding vaccination rate on the same date in the Hong Kong population of the same age and sex.

Of 14 167 patients, 8939 (61.2%) did not use oral antivirals; 5678 patients (39.8%) were prescribed antivirals, including 5195 (35.5%) who were given molnupiravir and 483 (3.3%) who were given nirmatrelvir/ritonavir. Compared with patients who did not use oral antivirals, patients taking molnupiravir and nirmatrelvir/ritonavir were more likely to be female and less likely to have comorbid illnesses (cardiovascular diseases, respiratory diseases, digestive diseases, chronic kidney diseases, diabetes, neurological diseases, and malignant tumors) and hospitalization in the past 1 year. Similar results were observed in the imputed data sets (eTable 3 in Supplement 1).

### Clinical Outcomes

At a median (IQR) follow-up of 30 (30-30) days, 6223 of 14 167 patients (42.6%) were hospitalized. Of them, 5332 (59.6%) did not use oral antivirals, 831 (16.0%) used molnupiravir, and 60 (12.4%) used nirmatrelvir/ritonavir. Of 2307 patients (15.8%) who experienced the secondary outcomes (ie, ICU admission, use of IMV, and/or death), 2102 (23.5%) did not use oral antivirals, 200 (3.8%) used molnupiravir, and 5 (1.0%) used nirmatrelvir/ritonavir.

### PS Weighting Analyses

After PS weighting, baseline clinical characteristics were more balanced among oral antiviral nonusers, molnupiravir users, and nirmatrelvir/ritonavir users (Table 2 and eTable 4 in Supplement 1). The use of molnupiravir (weighted hazard ratio [wHR], 0.46; 95% CI, 0.37-0.57; *P* < .001) and nirmatrelvir/ritonavir (wHR, 0.46; 95% CI, 0.32-0.65; *P* < .001) were both associated with a reduced risk of hospitalization. There was no significant difference between molnupiravir and nirmatrelvir/ritonavir users in terms of the risk of hospitalization (wHR, 1.00; 95% CI, 0.75-1.33; *P* = .99). The use of molnupiravir (wHR, 0.35; 95% CI, 0.23-0.51; *P* < .001) and nirmatrelvir/ritonavir (wHR, 0.17; 95% CI, 0.06-0.44; *P* < .001) were also associated with a reduced risk of the secondary outcomes (ICU admission, use of IMV, and/or death). There was no significant difference between molnupiravir and nirmatrelvir/ritonavir users for the risk of the secondary outcome (wHR, 0.49; 95% CI, 0.20-1.20; *P* = .12) (Table 3). A similar association between the use of COVID-19 oral antivirals and lower risk of the primary and secondary outcomes was observed in the 5-day landmark analysis (Table 3 and eFigure 2 in Supplement 1).

**Table 2.** Baseline Clinical Characteristics and Balancing Diagnostics Before and After Propensity Score Weighting for Patients With COVID-19, by Oral Antiviral Use

Characteristics	Before propensity score weighting^a^						After propensity score weighting^a^					
	No oral antivirals (n = 8939)	Molnupiravir (n = 5195)	Nirmatrelvir/ritonavir (n = 483)	ASMD^b^	ASMD^c^	ASMD^d^	No oral antivirals	Molnupiravir	Nirmatrelvir/ritonavir	ASMD^b^	ASMD^c^	ASMD^d^
Age, mean (SD), y	84.7 (10.4)	85.2 (9.8)	83.6 (10.1)	0.110	0.156	0.046	83.1 (10.8)	83.6 (10.4)	83.6 (10.1)	0.053	0.002	0.055
Male sex, No. (%)	4364 (48.8)	1849 (35.6)	182 (37.7)	0.230	0.043	0.273	189 (42.3)	184 (38.0)	182 (37.7)	0.095	0.006	0.089
Comorbidities, No. (%)												
Cardiovascular diseases	7027 (78.6)	3856 (74.2)	362 (74.9)	0.085	0.017	0.101	359 (80.2)	359 (74.2)	362 (74.9)	0.122	0.016	0.138
Digestive diseases	1583 (17.7)	361 (6.9)	29 (6.0)	0.493	0.040	0.453	24 (5.5)	30 (6.2)	29 (6.0)	0.022	0.010	0.032
Diabetes	3372 (37.7)	1786 (34.4)	151 (31.3)	0.139	0.067	0.072	150 (33.5)	149 (30.8)	151 (31.3)	0.049	0.011	0.060
Malignant tumor	449 (5.0)	60 (1.2)	9 (1.9)	0.234	0.052	0.286	17 (3.9)	8 (1.8)	9 (1.9)	0.150	0.008	0.158
Nervous system diseases	2053 (23.0)	263 (5.1)	18 (3.7)	1.016	0.071	0.945	22 (5.0)	18 (3.7)	18 (3.7)	0.068	0.002	0.069
Respiratory diseases	1392 (15.6)	240 (4.6)	18 (3.7)	0.625	0.047	0.578	26 (5.8)	17 (3.5)	18 (3.7)	0.111	0.013	0.123
Kidney diseases	1060 (11.9)	119 (2.3)	5 (1.0)	1.069	0.124	0.945	8 (1.7)	3 (0.7)	5 (1.0)	0.070	0.033	0.103
Laboratory parameters												
Hemoglobin, mean (SD), g/dL	11.5 (2.1)	11.8 (1.8)	12.1 (1.8)	0.344	0.203	0.147	11.9 (1.9)	12.1 (1.8)	12.1 (1.8)	0.080	0.019	0.099
White blood cell count mean (SD), cells/μL	8.9 (4.9)	7.5 (3.1)	7.7 (3.1)	0.385	0.049	0.433	7.5 (3.6)	7.6 (3.2)	7.7 (3.1)	0.020	0.008	0.028
Platelet count, mean (SD), ×10^3^/μL	244.0 (108.0)	243.2 (92.3)	246.3 (92.9)	0.025	0.033	0.003	254.3 (98.3)	242.9 (89.3)	246.3 (92.9)	0.103	0.022	0.125
Creatinine, median (IQR), mg/dL	0.99 (0.75-1.49)	0.88 (0.72-1.17)	0.85 (0.70-1.01)	2.265	0.776	0.956	0.80 (0.66-0.99)	0.81 (0.66-1.04)	0.85 (0.70-1.01)	0.021	0.046	0.067
Alanine aminotransferase, median (IQR), U/L	18 (12-28)	15 (11-22)	16 (11-23)	0.583	0.044	0.413	17 (12-26)	16 (11-23)	16 (11-23)	0.062	<0.001	0.063
Albumin, mean (SD), g/dL	3.09 (0.59)	3.41 (0.56)	3.64 (0.55)	0.978	0.411	0.559	3.51 (0.54)	3.62 (0.54)	3.64 (0.55)	0.193	0.017	0.210
Total bilirubin, mean (SD), mg/dL	0.59 (0.60)	0.55 (0.36)	0.59 (0.34)	0.010	0.121	0.135	0.57 (0.36)	0.59 (0.54)	0.59 (0.34)	0.029	0.038	0.067
Time from the first date of inclusion, mean (SD), d^e^	13.3 (10.6)	22.8 (9.7)	27.1 (9.9)	1.394	0.433	0.961	29.0 (10.9)	26.7 (8.7)	27.1 (9.9)	0.191	0.040	0.230
Hospitalizations in the past year, No. (%)												
0	5705 (63.8)	4507 (86.8)	414 (85.7)	0.626	0.030	0.655	328 (73.4)	425 (87.7)	414 (85.7)	0.353	0.058	0.411
1	1518 (17.0)	548 (10.5)	57 (11.8)	0.161	0.039	0.199	105 (23.4)	48 (9.9)	57 (11.8)	0.360	0.060	0.419
≥2	1716 (19.2)	140 (2.7)	12 (2.5)	1.074	0.014	1.060	14 (3.2)	12 (2.4)	12 (2.5)	0.048	0.006	0.054

Abbreviation: ASMD, absolute standardized mean difference.

SI conversion factors: To convert alanine aminotransferase to microkatals per liter, multiply by 0.0167; albumin to grams per liter, multiply by 10; bilirubin to micromoles per liter, multiply by 17.104; creatinine to micromoles per liter, multiply by 88.4; platelets to ×10^9 ^per liter, multiply by 1; white blood cells to ×10^9^ per liter, multiply by 0.001.

^a^ An ASMD less than 0.1 indicated a good balance between COVID-19 oral antiviral users and nonusers. Parameters with any ASMD 0.1 or higher would be adjusted in the doubly robust model. The weighted sample size after propensity score weighting was 447 nonusers, 484 molnupiravir users, and 483 nirmatrelvir/ritonavir users.

^b^ ASMD between COVID-19 oral antiviral nonusers and users of nirmatrelvir/ritonavir.

^c^ ASMD between users of molnupiravir and users of nirmatrelvir/ritonavir.

^d^ ASMD between users of molnupiravir and COVID-19 oral antiviral nonusers.

^e^ Days from the first date of inclusion referred to the number of days from the first date of inclusion (ie, February 16, 2022).

**Table 3.** Results of Weighted Cox Proportional Hazard Regression After Propensity Score Weighting for Patients With COVID-19 Who Received Care From Community Geriatric Assessment Team

COVID-19 oral antiviral use	Hospitalization		Death, ICU admission, or use of IMV	
	Weighted HR (95% CI)	*P* value	Weighted HR (95% CI)	*P* value
Main analysis, time-dependent analysis^a^				
No oral antiviral use as reference				
No oral antiviral use	1 [Reference]	NA	1 [Reference]	NA
Use of molnupiravir	0.46 (0.37-0.57)	<.001	0.35 (0.23-0.51)	<.001
Use of nirmatrelvir/ritonavir	0.46 (0.32-0.65)	<.001	0.17 (0.06-0.44)	<.001
Use of molnupiravir as reference				
No oral antiviral use	2.18 (1.74-2.73)	<.001	2.90 (1.95-4.31)	<.001
Use of molnupiravir	1 [Reference]	NA	1 [Reference]	NA
Use of nirmatrelvir/ritonavir	1.00 (0.75-1.33)	.99	0.49 (0.20-1.20)	.12
Sensitivity analysis, 5-d landmark analysis^b^				
No oral antiviral use as reference				
No oral antiviral use	1 [Reference]	NA	1 [Reference]	NA
Use of molnupiravir	0.31 (0.21-0.46)	<.001	0.28 (0.13-0.62)	.002
Use of nirmatrelvir/ritonavir	0.27 (0.16-0.46)	<.001	0.14 (0.03-0.71)	.02
Use of molnupiravir as reference				
No oral antiviral use	3.24 (2.19-4.81)	<.001	3.56 (1.61-7.90)	.002
Use of molnupiravir	1 [Reference]	NA	1 [Reference]	NA
Use of nirmatrelvir/ritonavir	0.88 (0.58-1.34)	.55	0.52 (0.12-2.14)	.37

Abbreviations: HR, hazard ratio; ICU, intensive care unit; IMV, invasive mechanical ventilation; NA, not applicable.

^a^ Use of molnupiravir and nirmatrelvir/ritonavir was modeled as a time-dependent covariate.

^b^ In the 5-day landmark analysis, patients were considered as molnupiravir and nirmatrelvir/ritonavir users if they received the treatment at baseline or within the first 5 days of follow-up; patients who experienced the clinical end points or were censored within the first 5 days of follow-up were excluded.

### Multivariable Analyses

The use of oral antivirals was associated with a reduced risk of hospitalization. Compared with patients who did not use oral antivirals, molnupiravir users (adjusted HR [aHR], 0.35; 95% CI, 0.32-0.38) and nirmatrelvir/ritonavir users (aHR, 0.31; 95% CI, 0.24-0.41) had a 65% and 69% lower risk of hospitalization, respectively (Table 4). The 5-day landmark analysis showed similar results of 69% and 76% for molnupiravir and nirmatrelvir/ritonavir users, respectively (eTable 5 in Supplement 1).

**Table 4.** Univariable and Multivariable Analysis by Cox Proportional Hazard Model on Factors Associated With Hospitalization in 14 617 Patients With COVID-19 Who Received Care From Community Geriatric Assessment Team^a^

Parameters	Univariable analysis		Multivariable analysis	
	HR (95% CI)	*P* value	aHR (95% CI)	*P* value
Use of COVID-19 oral antiviral^b^				
No oral antiviral use	1 [Reference]	NA	1 [Reference]	NA
Use of molnupiravir	0.28 (0.23-0.34)	<.001	0.35 (0.32-0.38)	<.001
Use of nirmatrelvir/ritonavir	0.23 (0.14-0.38)	<.001	0.31 (0.24-0.41)	<.001
Age	1.01 (1.01-1.01)	<.001	1.01 (1.01-1.02)	<.001
Male sex	1.34 (1.28-1.41)	<.001	1.21 (1.15-1.28)	<.001
Circulatory system disease	1.12 (1.06-1.19)	<.001	NA	NA
Digestive system disease	1.57 (1.47-1.68)	<.001	1.23 (1.15-1.32)	<.001
Diabetes	1.04 (0.99-1.09)	.15	NA	NA
Malignant tumor	1.54 (1.37-1.72)	<.001	NA	NA
Nervous system disease	1.88 (1.77-1.99)	<.001	1.47 (1.38-1.57)	<.001
Respiratory disease	1.74 (1.62-1.86)	<.001	1.29 (1.20-1.39)	<.001
Chronic kidney disease	1.95 (1.81-2.10)	<.001	NA	NA
Hemoglobin	0.95 (0.94-0.97)	<.001	1.02 (1.01-1.03)	.006
White blood cell count	1.05 (1.04-1.07)	<.001	1.04 (1.03-1.05)	<.001
Platelet count	0.99 (0.99-1.00)	.006	0.99 (0.99-0.99)	<.001
Creatinine	1.00 (1.00-1.00)	<.001	1.00 (1.00-1.00)	<.001
Alanine aminotransferase	1.00 (1.00-1.00)	<.001	1.00 (1.00-1.00)	<.001
Albumin	0.94 (0.94-0.95)	<.001	0.96 (0.96-0.97)	<.001
Total bilirubin	1.00 (1.00-1.01)	.005	NA	NA
Time from the first date of inclusion^c^	0.97 (0.97-0.98)	<.001	1.00 (1.00-1.01)	.04
Hospitalizations in the past year, No.				
0	1 [Reference]	NA	1 [Reference]	NA
1	0.60 (0.55-0.65)	<.001	0.46 (0.42-0.51)	<.001
≥2	1.00 (0.93-1.08)	.97	0.60 (0.55-0.65)	<.001

Abbreviations: aHR, adjusted subdistribution hazards ratio; HR, hazard ratio; NA, not applicable.

^a^ The use of COVID-19 oral antivirals was modeled as a time-dependent covariate. Use of COVID-19 oral antivirals referred to the use of molnupiravir or nirmatrelvir/ritonavir at baseline or during follow-up. Patients were followed from the baseline date to the last follow-up date (April 25, 2022), the date of first hospitalization, or the date of death, whichever came first.

^b^ Modeled as a time-dependent covariate.

^c^ Days from the first date of inclusion referred to the number of days from the first date of inclusion (ie, February 16, 2022).

Molnupiravir and nirmatrelvir/ritonavir were also associated with a lower risk of the secondary outcomes. Compared with patients who did not use oral antivirals, molnupiravir users (aHR, 0.38; 95% CI, 0.33-0.45) and nirmatrelvir/ritonavir users (aHR, 0.14; 95% CI, 0.06-0.35) had a lower risk of the secondary outcomes (Table 5). The 5-day landmark analysis indicated similar results for molnupiravir (aHR, 0.43; 95% CI, 0.34-0.53) and nirmatrelvir/ritonavir users (aHR, 0.13; 95% CI, 0.03-0.52) (eTable 6 in Supplement 1).

**Table 5.** Univariable and Multivariable Analysis by Cox Proportional Hazard Model on Factors Associated With Death, Intensive Care Unit Admission, and Use of Invasive Mechanical Ventilation in 14 617 Patients With COVID-19 Who Received Care From Community Geriatric Assessment Team^a^

Parameters	Univariable analysis		Multivariable analysis	
	HR (95% CI)	*P* value	aHR (95% CI)	*P* value
Use of COVID-19 oral antiviral^b^				
No oral antiviral use	1 [Reference]	NA	1 [Reference]	NA
Use of molnupiravir	0.20 (0.19-0.20)	<.001	0.38 (0.33-0.45)	<.001
Use of nirmatrelvir/ritonavir	0.06 (0.02-0.19)	<.001	0.14 (0.06-0.35)	<.001
Age	1.02 (1.02-1.03)	<.001	1.03 (1.03-1.04)	<.001
Male sex	1.62 (1.49-1.76)	<.001	1.47 (1.35-1.61)	<.001
Circulatory system disease	1.03 (0.93-1.13)	.56	0.84 (0.76-0.93)	<.001
Digestive system disease	1.56 (1.40-1.73)	<.001	1.14 (1.02-1.28)	.02
Diabetes	1.05 (0.97-1.14)	.24	NA	NA
Malignant tumor	1.81 (1.53-2.15)	<.001	1.35 (1.13-1.62)	.001
Nervous system disease	1.87 (1.70-2.06)	<.001	1.43 (1.30-1.58)	<.001
Respiratory disease	1.94 (1.75-2.15)	<.001	1.33 (1.19-1.49)	<.001
Chronic kidney disease	1.94 (1.73-2.18)	<.001	NA	NA
Hemoglobin	0.95 (0.93-0.98)	<.001	1.05 (1.02-1.07)	<.001
White blood cell count	1.06 (1.05-1.07)	<.001	1.04 (1.03-1.05)	<.001
Platelet count	0.99 (0.99-0.99)	<.001	0.99 (0.99-0.99)	<.001
Creatinine	1.00 (1.00-1.00)	<.001	1.00 (1.00-1.00)	<.001
Alanine aminotransferase	1.00 (1.00-1.00)	<.001	1.00 (1.00-1.00)	<.001
Albumin	0.92 (0.91-0.93)	<.001	0.94 (0.93-0.95)	<.001
Total bilirubin	1.01 (1.00-1.01)	<.001	NA	NA
Time from the first date of inclusion^c^	0.96 (0.95-0.96)	<.001	0.98 (0.98-0.98)	<.001
Hospitalizations in the past year, No.				
0	1 [Reference]	NA	1 [Reference]	NA
1	0.63 (0.55-0.73)	<.001	0.63 (0.54-0.73)	<.001
≥2	1.18 (1.05-1.32)	.006	0.81 (0.72-0.92)	.001

Abbreviations: aHR, adjusted subdistribution hazards ratio; HR, hazard ratio; NA, not applicable.

^a^ The use of COVID-19 oral antivirals was modeled as a time-dependent covariate. Use of COVID-19 oral antivirals refers to the use of molnupiravir or nirmatrelvir/ritonavir at baseline or during follow-up. Patients were followed from the baseline date to the last follow-up date (April 25, 2022), the date of first intensive care unit admission, the date of first use of invasive mechanical ventilation, or the date of death, whichever came first.

^b^ Modeled as a time-dependent covariate.

^c^ Days from the first date of inclusion referred to the number of days from the first date of inclusion (ie, February 16, 2022).

## Discussion

In this cohort study, we evaluated the association of 2 oral antivirals with hospitalization and inpatient disease progression for COVID-19 in patients living in nursing homes, who are the most vulnerable group of older patients. Our study is novel in showing that either molnupiravir or nirmatrelvir/ritonavir is associated with reduced risks of hospitalization and inpatient disease progression (ICU admission, use of IMV, and/or death). These findings are in keeping with those of 2 landmark RCTs.^5,6^ Of note, the present study is not a RCT comparing the 2 antivirals. A head-to-head direct comparison is warranted to compare their efficacy. It is very common that nursing home residents have clinical characteristics that are precautions or relative contraindications to oral antiviral use, such as chronic kidney diseases, dysphagia, and tube feeding. Physicians always exercise judicious judgment in prescribing oral antivirals, which are life-saving. The following discussion will explain the reasons for the differences in clinical characteristics between molnupiravir and nirmatrelvir/ritonavir users.

The Delta variant contributed to 58% and 98% of COVID-19 cases in the MOVe-OUT study^5^ and EPIC-HR study,^6^ respectively. Hong Kong has experienced the fifth wave of COVID-19, which is almost entirely caused by the Omicron variant, since December 31, 2021.^14^ As of September 8, 2022, 1 610 447 cases were reported.^15^ Of 9477 patients who died, more than one-half (54%) lived in nursing homes. Patients aged 60 years or older accounted for 82% and 96% of hospitalization and mortality, respectively.^15^ Thus, our study is a timely evaluation on the clinical effectiveness of oral antivirals against the Omicron variant, which is more infectious than the Delta variant, within the population who is most vulnerable to COVID-19–related morbidity and mortality.

Another recent retrospective cohort study^16^ in Hong Kong showed that molnupiravir reduced the risks of inpatient disease progression (ICU admission, use of IMV, and/or death) and death by 39% and 36%, respectively, but not the risk of hospitalization. Meanwhile, nirmatrelvir/ritonavir reduced the risks of hospitalization, inpatient disease progression, and death by 31%, 53%, and 75% respectively.^16^ There was no subgroup analysis on nursing home patients who contributed to 1.8% of 1 072 004 study patients.^16^ Arbel et al^17^ conducted a study on 109 254 patients aged 40 years and older in Israel and found that nirmatrelvir/ritonavir was effective in reducing the risks of hospitalization and death by 73% and 79%, respectively, in patients aged 65 years and older, but not the younger patients (aged 40-64 years). Nursing home patients were excluded.^17^ To add to this study, we have shown showed that nirmatrelvir was also associated with reduced risks of hospitalization and inpatient disease progression in nursing home patients. The findings of our work and these studies would have a substantial impact on clinical guidelines on treating nursing home patients with COVID-19.

Amid the peak of the fifth wave in the first quarter of 2022, the health care system of Hong Kong was overwhelmed by the high number of incident cases, reaching a peak of 52 000 to 55 000 cases per day between March 2 and March 4, 2022.^15^ In response, the government of Hong Kong refined the policy of treating nursing home residents with COVID-19. Conventionally, all nursing home patients with COVID-19, irrespective of its severity, would be hospitalized. As of February 17, 2022, patients with mild-to-moderate COVID-19 continued isolation and treatment in nursing homes with the support of CGAT.^18^ In March 2022, 2 oral antivirals were available in Hong Kong, and several community isolation facilities (CIFs) were set up. From March 28, 2022, onward, nursing home patients with mild-to-moderate COVID-19 were treated in the CIF. In Hong Kong, delivering intravenous and other parenteral treatments in nursing homes is difficult because of staffing constraints and lack of medical equipment. Thus, remdesivir and monoclonal antibodies are not feasible options.^19^ Together with medications for symptomatic relief, oral antivirals can be prescribed for nursing home patients as on-site treatment or before transfer to CIFs. The early outpatient use of oral antivirals can alleviate the huge demand for hospital beds and reduce mortality in the face of continuous outbreaks in the community and in nursing homes.

Our group has analyzed 2 cohorts of patients with COVID-19 during the same period of the Omicron wave in Hong Kong: community-dwelling patients (Yip et al^7^) and nursing home patients (the current study). Compared with their community-dwelling counterparts,^7^ nursing home patients were much older and had more comorbidities, including cardiovascular diseases, respiratory diseases, nervous system diseases, diabetes, kidney diseases, and malignant tumors. It is intriguing to compare the prescribing patterns between these 2 groups of patients. Of 93 883 community-dwelling patients attending designated clinics, 10 729 (11.4%) patients were prescribed antivirals, including molnupiravir for 5808 (54.1%) patients and nirmatrelvir/ritonavir for 4921 (45.9%) patients.^7^ Molnupiravir users were older and had more comorbidities compared with nirmatrelvir/ritonavir users. In contrast, of 14 617 nursing home patients in the current study, 5678 were prescribed with an antiviral, including molnupiravir for 5195 patients and nirmatrelvir/ritonavir for 483 patients. Molnupiravir and nirmatrelvir/ritonavir users had similar age and comorbidities. Nursing home patients were prescribed with oral antiviral therapy, mostly molnupiravir, more frequently than community-dwelling patients. There are 3 reasons. First, molnupiravir was available earlier than nirmatrelvir/ritonavir in March 2022. At the beginning, antivirals were restricted to patients with risk factors for disease progression, such as age 70 years and older, comorbidities (cardiovascular diseases, respiratory diseases, diabetes, and malignant tumor), and unvaccinated status. Second, with reference to the 2 landmark RCTs,^5,6^ although nirmatrelvir/ritonavir was more efficacious than molnupiravir in reducing hospitalization and death, nirmatrelvir/ritonavir is often contraindicated because of chronic kidney diseases, drug-drug interaction, and/or drug-disease interaction.^20,21^ Third, both nirmatrelvir and ritonavir are immediate-release film-coated tablets. Because of their uncertain efficacy and safety, nirmatrelvir/ritonavir tablets should not be chewed, broken, or crushed. They are, therefore, not recommended for patients with dysphagia or an enteral feeding tube, according to our local guidelines.^22,23^ In contrast, molnupiravir can be used for these patients by dissolving the contents of the capsule in water.^23,24^

The study by Yip et al^7^ did not demonstrate the benefits of molnupiravir in reducing the risk of hospitalization or the benefits of both antivirals in reducing the risk of inpatient disease progression. Yip et al^7^ attributed these findings to the first 2 aforementioned reasons. The other possible explanation was that the benefits of antivirals were diluted by the younger patients with fewer comorbidities. This rationale is supported by the findings of another recent Hong Kong study by Wong et al,^25^ which indicated that molnupiravir and nirmatrelvir/ritonavir were associated with reduced risks of mortality of stable hospitalized patients by 52% and 66%, respectively, and inpatient disease progression (ICU admission, use of IMV, and mortality) by 40% and 43%, respectively. However, subgroup analyses did not support substantial clinical benefits in patients younger than 65 years.^25^ The findings also echoed those of another study by Wong et al^16^ that molnupiravir reduced the risk of inpatient disease progression but not hospitalization. Summarizing all these findings in Hong Kong, oral antivirals would benefit older patients with multiple comorbidities. The findings of this study on nursing home patients can be reasonably extrapolated to other frail, community-dwelling older patients.

### Strengths and Limitations

The strengths of our study include a territory-wide cohort of patients with COVID-19 living in nursing homes. Our patients represented those encountered in daily clinical practice better than those enrolled in clinical trials.

Our study also has a few limitations. First, patients with COVID-19 who used oral antivirals had more comorbidities and past hospitalization. We compensated for this discrepancy by PS weighting and multivariable adjustment to balance the clinical characteristics in the patient groups. Second, the health care system was overwhelmed during the fifth wave of COVID-19 in Hong Kong, and this might have led to fewer hospitalizations than it should have.^18^ Still, some hospitalizations not related to COVID-19 might be included. We believe this would have affected patients prescribed and not prescribed with oral antivirals similarly if they were infected at the same time during the fifth wave. Therefore, the baseline date was balanced among the groups by PS weighting to reduce bias from varying hospitalization thresholds. Third, ascertainment bias may affect the reliability of study as the result of inaccurate entry of certain diagnosis codes for comorbidities. We minimized this bias by including laboratory and medication data for certain diagnoses such as diabetes and hypertension. Fourth, some laboratory parameters were missing, as in other retrospective studies. Multiple imputation was performed to reduce the selection bias. Fifth, the vaccination rate of nursing home patients was relatively low compared with other regions.^26^ Our findings may not be directly applicable to fully vaccinated patients.

## Conclusion

This territory-wide cohort study in Hong Kong reported the clinical effectiveness of 2 oral antivirals for treating COVID-19 among patients living in nursing homes. Both molnupiravir or nirmatrelvir/ritonavir were associated with reduced risks of hospitalization and inpatient disease progression. Given the ongoing outbreak worldwide, health authorities should allocate adequate resources for timely use of antiviral treatments for nursing home patients and other frail, community-dwelling older patients to reduce hospitalization and inpatient disease progression.

## Notes

Supplement 1. **eAppendix.** Supplemental Methods
eReferences
**eTable 1.** The Evolvement of the Treatment Guideline of COVID-19 Oral Antiviral Agents in Hong Kong Between 16 February 2022 and 31 March 2022 **eTable 2.** *International Classification of Diseases, Ninth Revision, Clinical Modification (ICD-9-CM)* Diagnosis and Procedure Codes for COVID-19 and Comorbidities **eTable 3.** Baseline Clinical Characteristics of the 14,617 COVID-19 Patients Who Received Care From Community Geriatric Assessment Team in 1 of the 20 Multiple Imputed Datasets **eTable 4.** Baseline Clinical Characteristics and Balancing Diagnostics Before and After Propensity Score Weighting in a Single Imputed Dataset in the 5-Day Landmark Analysis Between COVID-19 Patients Who Did Not Use Oral Antivirals, Used Molnupiravir, or Used Nirmatrelvir/Ritonavir (ie, Average Treatment Effect on the Patients Treated by Nirmatrelvir/Ritonavir) **eTable 5.** Univariate and Multivariable Analysis by Cox Proportional Hazard Model in the 5-Day Landmark Analysis on Factors Associated With Hospitalization in 14,617 COVID-19 Patients Who Received Care From Community Geriatric Assessment Team **eTable 6.** Univariate and Multivariable Analysis by Cox Proportional Hazard Model in the 5-Day Landmark Analysis on Factors Associated With Death/Intensive Care Unit (ICU) Admission/Use of Invasive Mechanical Ventilation (IMV) in 14,617 COVID-19 Patients Who Received Care From Community Geriatric Assessment Team **eFigure 1.** Patient Flowchart **eFigure 2.** Cumulative Incidence of (A) Hospitalization and (B) Death/Intensive Care Unit (ICU) Admission/Invasive Mechanical Ventilation (IMV) Use After Propensity Score Weighting in the 5-Day Landmark Analysis in a Single Imputed Dataset

Supplement 2.
Data Sharing Statement
