# Supplementary material for: Clinical Outcomes Following Treatment for COVID-19 With Nirmatrelvir/Ritonavir and Molnupiravir Among Patients Living in Nursing Homes
Source: JAMA Netw Open. 2023 Apr 27;6(4):e2310887. doi: 10.1001/jamanetworkopen.2023.10887 (PMC10140804; doi:10.1001/jamanetworkopen.2023.10887)
Supplement: Supplement 1. — eAppendix. Supplemental Methods eReferences eTable 1. The Evolvement of the Treatment Guideline of COVID-19 Oral Antiviral Agents in Hong Kong Between 16 February 2022 and 31 March 2022 eTable 2. International Classification of Diseases, Ninth Revision, Clinical Modification (ICD-9-CM) Diagnosis and Procedure Codes for COVID-19 and Comorbidities eTable 3. Baseline Clinical Characteristics of the 14,617 COVID-19 Patients Who Received Care From Community Geriatric Assessment Team in 1 of the 20 Multiple Imputed Datasets eTable 4. Baseline Clinical Characteristics and Balancing Diagnostics Before and After Propensity Score Weighting in a Single Imputed Dataset in the 5-Day Landmark Analysis Between COVID-19 Patients Who Did Not Use Oral Antivirals, Used Molnupiravir, or Used Nirmatrelvir/Ritonavir (ie, Average Treatment Effect on the Patients Treated by Nirmatrelvir/Ritonavir) eTable 5. Univariate and Multivariable Analysis by Cox Proportional Hazard Model in the 5-Day Landmark Analysis on Factors Associated With Hospitalization in 14,617 COVID-19 Patients Who Received Care From Community Geriatric Assessment Team eTable 6. Univariate and Multivariable Analysis by Cox Proportional Hazard Model in the 5-Day Landmark Analysis on Factors Associated With Death/Intensive Care Unit (ICU) Admission/Use of Invasive Mechanical Ventilation (IMV) in 14,617 COVID-19 Patients Who Received Care From Community Geriatric Assessment Team eFigure 1. Patient Flowchart eFigure 2. Cumulative Incidence of (A) Hospitalization and (B) Death/Intensive Care Unit (ICU) Admission/Invasive Mechanical Ventilation (IMV) Use After Propensity Score Weighting in the 5-Day Landmark Analysis in a Single Imputed Dataset [file jamanetwopen-e2310887-s001.pdf]

Supplemental Online Content

Ma BHM, Yip TCF, Lui GCY, et al. Clinical outcomes following treatment for COVID-19 with nirmatrelvir/ritonavir and molnupiravir among patients living in nursing homes. *JAMA Netw Open*. 2023;6(4):e2310887. doi:10.1001/jamanetworkopen.2023.10887

eAppendix. Supplemental Methods
eReferences
eTable 1. The Evolvement of the Treatment Guideline of COVID-19 Oral Antiviral Agents in Hong Kong Between 16 February 2022 and 31 March 2022
eTable 2. *International Classification of Diseases, Ninth Revision, Clinical Modification (ICD-9-CM)* Diagnosis and Procedure Codes for COVID-19 and Comorbidities
eTable 3. Baseline Clinical Characteristics of the 14,617 COVID-19 Patients Who Received Care From Community Geriatric Assessment Team in 1 of the 20 Multiple Imputed Datasets
eTable 4. Baseline Clinical Characteristics and Balancing Diagnostics Before and After Propensity Score Weighting in a Single Imputed Dataset in the 5-Day Landmark Analysis Between COVID-19 Patients Who Did Not Use Oral Antivirals, Used Molnupiravir, or Used Nirmatrelvir/Ritonavir (ie, Average Treatment Effect on the Patients Treated by Nirmatrelvir/Ritonavir)
eTable 5. Univariate and Multivariable Analysis by Cox Proportional Hazard Model in the 5-Day Landmark Analysis on Factors Associated With Hospitalization in 14,617 COVID-19 Patients Who Received Care From Community Geriatric Assessment Team
eTable 6. Univariate and Multivariable Analysis by Cox Proportional Hazard Model in the 5-Day Landmark Analysis on Factors Associated With Death/Intensive Care Unit (ICU) Admission/Use of Invasive Mechanical Ventilation (IMV) in 14,617 COVID-19 Patients Who Received Care From Community Geriatric Assessment Team
eFigure 1. Patient Flowchart
eFigure 2. Cumulative Incidence of (A) Hospitalization and (B) Death/Intensive Care Unit (ICU) Admission/Invasive Mechanical Ventilation (IMV) Use After Propensity Score Weighting in the 5-Day Landmark Analysis in a Single Imputed Dataset

This supplemental material has been provided by the authors to give readers additional information about their work.

© 2023 Ma BHM et al. *JAMA Network Open.*

eAppendix. Supplemental Methods

Details on definitions

The diagnosis code of COVID-19 (519.8:8) was adopted in public hospitals and clinics in Hong Kong under the Hospital Authority Master Disease Code Table (HAMDCT). The HAMDCT extends the ICD-9-CM system with additional, locally relevant terms and associated codes. A code number of 519.8:8 was added to the HAMDCT, indicating that COVID-19 was present.[1] Significant comorbidities were defined as follows: hypertension was identified by any use of anti-hypertensive drugs and/or ICD-9-CM diagnosis codes (401-405); diabetes mellitus was defined by exposure to any anti-diabetic agents, and/or hemoglobin A_{1c} ≥6.5%, and/or fasting plasma glucose ≥7 mmol/L, and/or the ICD-9-CM diagnosis codes (250.00-250.93).[2] Other comorbidities were identified based on ICD-9-CM codes (Supplementary Table 2).

Details on multiple imputation

Before estimating the propensity score, missing data were assumed missing at random and replaced by multiple imputation by chained equations to create twenty complete data sets after ten initial burn-in iterations.[3,4] The imputed baseline variables (missing percentage) were hemoglobin (4.6%), white blood cell counts (4.6%), platelet counts (4.6%), creatinine (2.8%), alanine aminotransferase (4.2%), albumin (4.4%), and total bilirubin (4.5%). The variables included in the imputation models were all covariates included in PS estimation, the occurrence of primary endpoint, and the corresponding Nelson-Aalen estimator of the cumulative hazard at the time of event or censoring; the geographical clusters of the nursing home was included as cluster in the 2-level multiple imputation.[5] All imputed values were constrained within plausible ranges.

Details on Cox proportional hazard regression and Kaplan-Meier method

Cumulative incidence with 95% confidence interval of the primary and secondary endpoints of the three groups was estimated by Kaplan-Meier method. Besides PS weighting analysis, we performed univariate and multivariable analyses by Cox proportional hazards regression; backward stepwise selection was performed to select significant covariates on multivariable analysis. Weighted Cox proportional hazards regression was used in the PS weighting analysis. Clinical characteristics with ASMD ≥0.2 after PS weighting were included in the weighted Cox model for doubly robust adjustment. Separate baseline hazards were estimated for each of the seven geographical clusters of nursing home. Cluster-robust variance estimates were used to calculate 95% CIs.[6] The overall coefficient estimates and standard errors were computed by combining the estimates obtained on each multiple imputation data set using Rubin's rules.[7] Schoenfeld residual plots were used to assess the proportional hazards assumption, which did not detect any significant violations.

Details on time-dependent covariate and 5-day landmark analysis

To tackle immortal time bias arising from the initiation of molnupiravir and nirmatrelvir/ritonavir during follow-up, use of molnupiravir and nirmatrelvir/ritonavir was modeled as a time-dependent covariate. As a sensitivity analysis, a 5-day landmark analysis was performed to tackle immortal time bias instead of time-dependent analysis. In the 5-day landmark analysis, patients were considered as molnupiravir and nirmatrelvir/ritonavir users if they received the treatment at baseline or within the first 5 days of follow-up; patients who developed the clinical end-points or censored within the first 5 days of follow-up were excluded. A 5-day landmark was chosen to observe the use of oral antivirals and allow a complete treatment course of the majority of patients who received the oral antivirals on the first day. Meanwhile, patients without oral antiviral use would survive for at least 5 days to avoid the immortal time in patients receiving antiviral treatment who were likely to finish the 5-day treatment course.

© 2023 Ma BHM et al. *JAMA Network Open.*

eReferences

1. Wai AKC, Wong CKH, Wong JYH, et al. Changes in Emergency Department Visits, Diagnostic Groups, and 28-Day Mortality Associated With the COVID-19 Pandemic: A Territory-Wide, Retrospective, Cohort Study. *Ann Emerg Med*. Feb 2022;79(2):148-157. doi:10.1016/j.annemergmed.2021.09.424

2. American Diabetes Association Professional Practice Committee. 2. Classification and Diagnosis of Diabetes: Standards of Medical Care in Diabetes-2022. *Diabetes Care*. Jan 1 2022;45(Suppl 1):S17-S38. doi:10.2337/dc22-S002

3. Hamilton BH, Ko CY, Richards K, Hall BL. Missing data in the American College of Surgeons National Surgical Quality Improvement Program are not missing at random: implications and potential impact on quality assessments. Research Support, Non-U.S. Gov't. *J Am Coll Surg*. Feb 2010;210(2):125-139 e2. doi:10.1016/j.jamcollsurg.2009.10.021

4. Little RJ, D'Agostino R, Cohen ML, et al. The prevention and treatment of missing data in clinical trials. *N Engl J Med*. Oct 4 2012;367(14):1355-60. doi:10.1056/NEJMsr1203730

5. White IR, Royston P. Imputing missing covariate values for the Cox model. Research Support, Non-U.S. Gov't. *Stat Med*. Jul 10 2009;28(15):1982-98. doi:10.1002/sim.3618

6. Austin PC. The use of propensity score methods with survival or time-to-event outcomes: reporting measures of effect similar to those used in randomized experiments. Research Support, Non-U.S. Gov't. *Stat Med*. Mar 30 2014;33(7):1242-58. doi:10.1002/sim.5984

7. Rubin DB, Schenker N. Multiple imputation in health-care databases: an overview and some applications. Research Support, U.S. Gov't, Non-P.H.S.
Research Support, U.S. Gov't, P.H.S.
Review. *Stat Med*. Apr 1991;10(4):585-98. doi:10.1002/sim.4780100410

© 2023 Ma BHM et al. *JAMA Network Open.*

eTable 1. The Evolvement of the Treatment Guideline of COVID-19 Oral Antiviral Agents in Hong Kong Between 16 February 2022 and 31 March 2022

Period	Recommendation	Treatment Indication
Before 2 March 2022[a]	Not applicable	Not applicable
2 March 2022 – 4 March 2022	Molnupiravir[b] only	Nosocomial infection + unvaccinated + (age ≥70 OR age <70 with high risk factors) AND within 5 days of symptoms AND Ct <25 AND SpO2 >94%
5 March 2022 – 10 March 2022	Molnupiravir[b] only	(nosocomial infection OR institutional transmission) + unvaccinated + (age ≥70 OR age <70 with high risk factors) AND within 5 days of symptoms AND RAT/PCR positive AND SpO2 >94%
11 March 2022 – 20 March 2022	Molnupiravir[b] AND nirmatrelvir/ritonavir[c]	Incomplete vaccination + (Age ≥70 OR age <70 with high risk factors) AND within 5 days of symptoms AND RAT/PCR positive AND SpO2 >94%
21 March 2022 onward	Nirmatrelvir/ritonavir (preferred)[c] or Molnupiravir[b]	Age ≥60 OR (age <60 with high risk factors + incomplete vaccination) OR severely immunocompromised, AND within 5 days of symptoms AND RAT/PCR positive AND SpO2 >94%

[a] Both molnupiravir and nirmatrelvir/ritonavir were not available in Hong Kong before 2 March 2022.
[b] Molnupiravir is contraindicated during pregnancy and lactation.
[c] Nirmatrelvir/ritonavir is also contraindicated in patients with impaired renal function (eGFR <30 mL/min/1.73m^2), in those on nasogastric tube feeding, those with swallowing difficulties who cannot swallow whole tablets, and those on the following medications:
Examples of significant/potentially significant drug interactions [consult full prescribing information if needed]

- Alpha1-adrenoreceptor antagonist: alfuzosin
- Analgesics: pethidine, propoxyphene
- Antiarrhythmic: amiodarone, dronedarone, flecainide, propafenone, quinidine
- Antimycobacterial: rifampicin
- Anticancer drug: apalutamide
- Anticoagulants: warfarin, rivaroxaban
- Anticonvulsant: carbamazepine, phenobarbital, phenytoin
- Anti-gout: colchicine
- Antipsychotics: lurasidone, pimozide, clozapine
- Cardiac glycosides: digoxin
- HMG-CoA reductase inhibitors: atorvastatin, lovastatin, rosuvastatin, simvastatin
- Hormonal contraceptive: ethinyl estradiol
- PDE5 inhibitor: sildenafil when used for pulmonary arterial hypertension

Abbreviation: Ct = cycle threshold, eGFR = estimated glomerular filtration rate, PCR = polymerase chain reaction, RAT = rapid antigen test, SpO2 = peripheral oxygen saturation.

© 2023 Ma BHM et al. *JAMA Network Open.*

eTable 2. ***International Classification of Diseases, Ninth Revision, Clinical Modification (ICD-9-CM)*** **Diagnosis and Procedure Codes for COVID-19 and Comorbidities**

Disease	ICD-9-CM Code	Description
COVID-19	519.8:8	COVID-19
Cardiovascular diseases		
Hypertension and hypertensive diseases	401<br>402<br>403<br>404<br>405	Essential hypertension<br>Hypertensive heart disease<br>Hypertensive chronic kidney disease<br>Hypertensive heart and chronic kidney disease<br>Secondary hypertension
Ischemic heart disease	410<br>411<br>412<br>413<br>414	Acute myocardial infarction<br>Other acute and subacute forms of ischemic heart disease<br>Old myocardial infarction<br>Angina pectoris<br>Other forms of chronic ischemic heart disease
Cardiac dysrhythmias	427	Cardiac dysrhythmias
Heart failure	428	Heart failure
Digestive diseases		
Peptic ulcer	530.2<br>531<br>532<br>533<br>534	Ulcer of esophagus<br>Gastric ulcer<br>Duodenal ulcer<br>Peptic ulcer site unspecified<br>Gastrojejunal ulcer
Chronic liver disease, liver failure, liver cirrhosis and complications	070.2-3<br>070.41, 44<br>070.51, 54<br>V02.61<br>V02.62<br>070.42, 52<br>275.0<br>275.1<br>273.4<br>570<br>571<br>572<br>573.0-5<br>348.3<br>349.82<br>456.0, 20<br>456.1, 21<br>456.8:1-2<br>456.8:4-5<br>567.2:9<br>789.5	Chronic hepatitis B<br>Hepatitis C with hepatic coma<br>Hepatitis C without mention of hepatic coma<br>Hepatitis B carrier<br>Hepatitis C carrier<br>Hepatitis delta without mention of active hepatitis B<br>Hemochromatosis<br>Wilson's disease<br>Alpha-1 antitrypsin disease<br>Acute and subacute necrosis of liver<br>Chronic liver disease and cirrhosis<br>Liver abscess and sequelae of chronic liver disease<br>Other disorders of liver<br>Encephalopathy, unspecified<br>Toxic encephalopathy<br>Esophageal varices with bleeding<br>Esophageal varices without bleeding<br>Bleeding gastric varices<br>Gastric varices<br>Spontaneous bacterial peritonitis<br>Ascites
Biliary disease	574<br>575<br>576	Cholelithiasis<br>Disorders of gallbladder<br>Disorders of biliary tract
Gastrointestinal hemorrhage	578	Gastrointestinal hemorrhage
Diabetes mellitus		
Diabetes mellitus	250	Diabetes mellitus

© 2023 Ma BHM et al. *JAMA Network Open.*

Disease	ICD-9-CM Code	Description
Malignant tumor		
Malignant neoplasm	140-149<br>150-159<br>160-165<br>170-176<br>179-189<br>190-199<br>200-209<br>235-238<br>239	Malignant neoplasm of lip, oral cavity, and pharynx<br>Malignant neoplasm of digestive organs and peritoneum<br>Malignant neoplasm of respiratory and intrathoracic organ<br>Malignant neoplasm of bone, connective tissue, skin, and breast<br>Malignant neoplasm of genitourinary organs<br>Malignant neoplasm of other and unspecified sites<br>Malignant neoplasm of lymphatic and hematopoietic tissue<br>Neoplasms of uncertain behavior<br>Neoplasms of unspecified nature
Chemotherapy	V58.1<br>V66.2<br>V67.2<br>99.25	Encounter for antineoplastic chemotherapy and immunotherapy<br>Convalescence following chemotherapy<br>Follow-up examination, following chemotherapy<br>Injection or infusion of cancer chemotherapeutic substance
History of cancer	V10	Personal history of cancer
Nervous system diseases		
Cerebrovascular events	430<br>431<br>432<br>433<br>434<br>435<br>436<br>437<br>438	Subarachnoid hemorrhage<br>Intracerebral hemorrhage<br>Other and unspecified intracranial hemorrhage<br>Occlusion and stenosis of precerebral arteries<br>Occlusion of cerebral arteries<br>Transient cerebral ischemia<br>Acute, but ill-defined, cerebrovascular disease<br>Other and ill-defined cerebrovascular disease<br>Late effects of cerebrovascular disease
Other nervous system disease	320-327<br>330-337<br>340-345	Inflammatory diseases of the central nervous system<br>Hereditary and degenerative diseases of the central nervous system<br>Other disorders of the central nervous system
Respiratory diseases		
Chronic obstructive pulmonary disease and allied conditions	490-496	Chronic obstructive pulmonary disease and allied conditions
Pneumoconioses and other lung diseases due to external agents	500-508	Pneumoconioses and other lung diseases due to external agents
Other diseases of respiratory system	510-519	Other diseases of respiratory system
Kidney diseases		
Nephritis, nephrotic syndrome, and nephrosis	581<br>582<br>583<br>584<br>585<br>586<br>587	Nephrotic syndrome<br>Chronic glomerulonephritis<br>Nephritis and nephropathy not specified as acute or chronic<br>Acute kidney failure<br>Chronic kidney disease<br>Renal failure, unspecified

© 2023 Ma BHM et al. *JAMA Network Open.*

Disease	ICD-9-CM Code	Description
	588	Renal sclerosis, unspecified<br>Disorders resulting from impaired renal function
Renal replacement therapy	V56	Encounter for dialysis and dialysis catheter care
	38.95	Venous catheterization for renal dialysis
	39.27	Arteriovenostomy for renal dialysis
	39.42	Revision of arteriovenous shunt for renal dialysis
	39.43	Removal of arteriovenous shunt for renal dialysis
	39.95	Hemodialysis
	54.98	Peritoneal dialysis
Human immunodeficiency virus (HIV)		
HIV	042	HIV disease
HIV	079.53	HIV, type 2 [HIV-2]
HIV	V02.9:1	HIV carrier
HIV	V08	Asymptomatic HIV infection status

ICD-9-CM = International Classification of Diseases, Ninth Revision, Clinical Modification.

© 2023 Ma BHM et al. *JAMA Network Open.*

eTable 3. Baseline Clinical Characteristics of the 14,617 COVID-19 Patients Who Received Care From Community Geriatric Assessment Team in 1 of the 20 Multiple Imputed Datasets

Clinical characteristics	All N=14,617	COVID-19 oral anti-viral non-user N=8,939	Use of molnupiravir N=5,195	Use of nirmatrelvir/ ritonavir N=483	*P* value
**Age (years)**	84.8 ± 10.2	84.7 ± 10.4	85.2 ± 9.8	83.6 ± 10.1	<0.001
**Male gender (n, %)**	6,395 (43.8)	4,364 (48.8)	1,849 (35.6)	182 (37.7)	<0.001
**Co-morbidities (n, %)**[a]					
**Cardiovascular diseases**	11,245 (76.9)	7,027 (78.6)	3,856 (74.2)	362 (74.9)	<0.001
- Hypertension	11,027 (75.4)	6,852 (76.7)	3,816 (73.5)	359 (74.3)	<0.001
- Ischemic heart disease	774 (5.3)	686 (7.7)	86 (1.7)	2 (0.4)	<0.001
- Cardiac dysrhythmias	1,470 (10.1)	1,180 (13.2)	277 (5.3)	13 (2.7)	<0.001
- Heart failure	847 (5.8)	724 (8.1)	120 (2.3)	3 (0.6)	<0.001
**Digestive diseases**	1,973 (13.5)	1,583 (17.7)	361 (6.9)	29 (6.0)	<0.001
- Peptic ulcer	225 (1.5)	197 (2.2)	26 (0.5)	2 (0.4)	<0.001
- Chronic liver disease	882 (6.0)	626 (7.0)	235 (4.5)	21 (4.3)	<0.001
- Liver failure, cirrhosis, or cirrhotic complications	29 (0.2)	28 (0.3)	1 (0.0)	0 (0.0)	<0.001
- Biliary disease	372 (2.5)	332 (3.7)	40 (0.8)	0 (0.0)	<0.001
- Gastrointestinal hemorrhage	773 (5.3)	676 (7.6)	88 (1.7)	9 (1.9)	<0.001
**Diabetes mellitus**	5,309 (36.3)	3,372 (37.7)	1,786 (34.4)	151 (31.3)	<0.001
**Malignant tumors**	518 (3.5)	449 (5.0)	60 (1.2)	9 (1.9)	<0.001
**Nervous system diseases**	2,334 (16.0)	2,053 (23.0)	263 (5.1)	18 (3.7)	<0.001
- Cerebrovascular events	1,687 (11.5)	1,494 (16.7)	184 (3.5)	9 (1.9)	<0.001
- Other nervous system diseases	998 (6.8)	878 (9.8)	110 (2.1)	10 (2.1)	<0.001
**Respiratory diseases**	1,650 (11.3)	1,392 (15.6)	240 (4.6)	18 (3.7)	<0.001
**Kidney diseases**	1,184 (8.1)	1,060 (11.9)	119 (2.3)	5 (1.0)	<0.001
**HIV infection**	13 (0.1)	9 (0.1)	2 (0.0)	2 (0.4)	0.04
**Laboratory parameters**					
**Hemoglobin (g/dL)**	11.6 ± 2.0	11.5 ± 2.1	11.8 ± 1.8	12.0 ± 1.8	<0.001
**White blood cell (x10^9/L)**	8.4 ± 4.4	8.9 ± 4.9	7.6 ± 3.2	7.6 ± 3.0	<0.001
**Platelet (x10^9/L)**	243.6 ± 102.2	243.7 ± 108.0	243.4 ± 92.4	244.9 ± 91.6	0.95
**Creatinine (μmol/L)**	83 (64-119)	88 (66-132)	78 (62-104)	75 (60-89)	<0.001
**Alanine aminotransferase (U/L)**	17 (11-26)	18 (12-29)	15 (10-22)	16 (11-24)	<0.001
**Albumin (g/L)**	32.2 ± 6.0	30.9 ± 5.9	34.1 ± 5.6	36.1 ± 5.6	<0.001
**Total bilirubin (μmol/L)**	9.9 ± 9.0	10.2 ± 10.3	9.3 ± 6.5	10.0 ± 6.3	<0.001
**Days from the first date of inclusion**[b]	17.1 ± 11.3	13.3 ± 10.6	22.8 ± 9.7	27.1 ± 9.9	<0.001

© 2023 Ma BHM et al. *JAMA Network Open.*

Clinical characteristics	All N=14,617	COVID-19 oral anti-viral non-user N=8,939	Use of molnupiravir N=5,195	Use of nirmatrelvir/ ritonavir N=483	*P* value
**Age- and sex-specified complete vaccination rate (%)[c]**	20.9 ± 12.8	20.4 ± 12.8	21.5 ± 12.6	24.1 ± 13.7	<0.001
**Number of hospitalizations in the past year (n, %)**					<0.001
- 0	10,626 (72.7)	5,705 (63.8)	4,507 (86.8)	414 (85.7)	
- 1	2,123 (14.5)	1,518 (17.0)	548 (10.5)	57 (11.8)	
- ≥2	1,868 (12.8)	1,716 (19.2)	140 (2.7)	12 (2.5)	
**Follow-up duration (days)**	30 (30-30)	30 (28-30)	30 (30-30)	30 (30-30)	<0.001

All co-morbidities were represented as binary parameters.
Categorical variables were presented as number (percentage). Follow-up duration was expressed in median (25th percentile - 75th percentile). Age was expressed in mean ± standard deviation. Qualitative and quantitative differences between subgroups were analyzed by Chi-square or Fisher's exact tests for categorical parameters and Student's t test or Mann-Whitney U test for continuous parameters, as appropriate.

[a] The definition of co-morbidities was stated in Supplementary Table 2.

[b] Days from the first date of inclusion referred to the number of days from the first date of inclusion, *i.e.* 16 February 2022.

[c] Age- and sex-specified complete vaccination rate referred to the corresponding vaccination rate on the same date in the Hong Kong population of the same age and gender.

© 2023 Ma BHM et al. *JAMA Network Open.*

eTable 4. Baseline Clinical Characteristics and Balancing Diagnostics Before and After Propensity Score Weighting in a Single Imputed Dataset in the 5-Day Landmark Analysis Between COVID-19 Patients Who Did Not Use Oral Antivirals, Used Molnupiravir, or Used Nirmatrelvir/Ritonavir (ie, Average Treatment Effect on the Patients Treated by Nirmatrelvir/Ritonavir)

	Before propensity score weighting						After propensity score weighting					
Clinical characteristics	COVID-19 oral anti-viral non-user N=9,827	Use of molnupiravir N=3,158	Use of nirmatrelvir/ ritonavir N=282	ASMD[a]	ASMD[b]	ASMD[c]	COVID-19 oral anti-viral non-user	Use of molnupiravir	Use of nirmatrelvir/ ritonavir	ASMD[a]	ASMD[b]	ASMD[c]
Age (years)	84.8±10.3	85.1±9.6	83.6±10.5	0.109	0.139	0.030	82.4±10.4	83.8±10.2	83.6±10.5	0.120	0.013	0.133
Male sex (n, %)	4,419 (45.0)	1,172 (37.1)	105 (37.2)	0.160	0.003	0.163	274 (44.6)	97 (36.6)	105 (37.2)	0.152	0.013	0.165
**Co-morbidities (n, %)**												
Cardiovascular diseases	7,662 (78.0)	2,319 (73.4)	214 (75.9)	0.049	0.057	0.106	493 (80.2)	200 (75.0)	214 (75.9)	0.101	0.020	0.122
Digestive diseases	1,488 (15.1)	209 (6.6)	14 (5.0)	0.469	0.076	0.392	21 (3.4)	13 (4.9)	14 (5.0)	0.074	0.004	0.070
Diabetes mellitus	3,666 (37.3)	1,051 (33.3)	91 (32.3)	0.108	0.022	0.086	212 (34.5)	82 (30.7)	91 (32.3)	0.047	0.034	0.081
Malignant tumor	415 (4.2)	39 (1.2)	6 (2.1)	0.145	0.062	0.207	32 (5.2)	4 (1.5)	6 (2.1)	0.214	0.046	0.260
Nervous system diseases	1,846 (18.8)	128 (4.1)	6 (2.1)	1.154	0.133	1.021	19 (3.2)	4 (1.4)	6 (2.1)	0.071	0.050	0.121
Respiratory diseases	1,297 (13.2)	132 (4.2)	8 (2.8)	0.624	0.081	0.543	24 (4.0)	6 (2.2)	8 (2.8)	0.068	0.041	0.109
Kidney diseases	948 (9.6)	65 (2.1)	1 (0.4)	1.563	0.287	1.277	3 (0.5)	0 (0.1)	1 (0.4)	0.019	0.037	0.056
**Laboratory parameters**												
Hemoglobin (g/dL)	11.5±2.0	11.8±1.8	12.0±1.8	0.280	0.153	0.127	11.8±1.9	12.1±1.8	12.0±1.8	0.147	0.031	0.179
White blood cell (x10^9/L)	8.5±4.5	7.5±3.2	7.9±3.0	0.217	0.113	0.330	7.8±4.1	7.8±3.3	7.9±3.0	0.040	0.020	0.020
Platelet (x10^9/L)	247.4±106.3	245.8±91.3	248.4±91.5	0.012	0.028	0.017	256.6±83.1	247.1±85.3	248.4±91.5	0.090	0.014	0.104
Creatinine (µmol/L)	85 (64-123)	77 (62-103)	75 (62-88)	1.358	0.484	0.874	69 (56-86)	72 (59-93)	75 (62-88)	0.201	0.019	0.182
ALT (U/L)	17 (12-27)	15 (10-23)	17 (11-27)	0.320	0.009	0.329	17 (12-26)	15 (11-23)	17 (11-27)	0.069	0.008	0.061
Albumin (g/L)	31.6±5.9	34.3±5.7	36.6±5.9	0.845	0.389	0.456	35.5±5.5	36.9±5.4	36.6±5.9	0.181	0.049	0.230
Total bilirubin (µmol/L)	10.1±9.4	9.4±6.9	9.6±5.4	0.083	0.044	0.128	9.2±6.0	9.6±7.0	9.6±5.4	0.073	0.009	0.064
Days from the first date of inclusion[d]	13.3±10.4	27.6±5.8	33.0±4.1	4.830	1.323	3.507	37.3±5.4	32.2±6.2	33.0±4.1	1.042	0.208	1.250
Number of hospitalizations in the past year (n, %)												
- 0	6,711 (68.3)	2,758 (87.3)	243 (86.2)	0.518	0.034	0.552	495 (80.6)	243 (91.2)	243 (86.2)	0.162	0.145	0.307
- 1	1,574 (16.0)	332 (10.5)	33 (11.7)	0.134	0.037	0.171	108 (17.6)	20 (7.6)	33 (11.7)	0.185	0.128	0.313
- ≥2	1,542 (15.7)	68 (2.2)	6 (2.1)	0.940	0.002	0.938	11 (1.8)	3 (1.3)	6 (2.1)	0.022	0.060	0.038

Use of COVID-19 oral anti-viral referred to the use of molnupiravir or nirmatrelvir/ritonavir at baseline or during follow-up. 63.6% of the COVID-19 oral anti-viral users used the anti-viral drugs within the first 5 days of follow-up. An ASMD <0.2 indicated a good balance between COVID-19 oral anti-viral users and non-users. Parameters with ASMD ≥0.2 would be adjusted in the doubly robust model.

The weighted sample size after propensity score weighting was 617, 266, and 282 in non-users, molnupiravir users, and nirmatrelvir/ritonavir users respectively.

[a] ASMD between COVID-19 oral anti-viral non-users and users of nirmatrelvir/ritonavir.

[b] ASMD between users of molnupiravir and users of nirmatrelvir/ritonavir.

[c] ASMD between users of molnupiravir and COVID-19 oral anti-viral non-users.

[d] Days from the first date of inclusion referred to the number of days from the first date of inclusion, *i.e.,* 16 February 2022.

ALT = Alanine aminotransferase, ASMD = absolute standardized mean difference, SARS-CoV-2 = severe acute respiratory syndrome coronavirus 2.

© 2023 Ma BHM et al. *JAMA Network Open.*

eTable 5. Univariate and Multivariable Analysis by Cox Proportional Hazard Model in the 5-Day Landmark Analysis on Factors Associated With Hospitalization in 14,617 COVID-19 Patients Who Received Care From Community Geriatric Assessment Team

Parameters	Univariate analysis		Multivariable analysis	
	**HR (95% CI)**	***P* value**	**aHR (95% CI)**	***P* value**
Use of COVID-19 oral anti-viral				
- No oral anti-viral use	Referent		Referent	
- Use of molnupiravir	0.24 (0.22-0.27)	<0.001	0.31 (0.28-0.35)	<0.001
- Use of nirmatrelvir/ritonavir	0.16 (0.11-0.24)	<0.001	0.24 (0.16-0.35)	<0.001
Age (years)	1.01 (1.01-1.02)	<0.001	1.01 (1.01-1.02)	<0.001
Male gender	1.33 (1.26-1.41)	<0.001	1.26 (1.19-1.34)	<0.001
Circulatory system disease	1.14 (1.06-1.22)	<0.001		
Digestive system disease	1.57 (1.46-1.69)	<0.001	1.25 (1.16-1.34)	<0.001
Diabetes mellitus	1.04 (0.98-1.10)	0.18		
Malignant tumor	1.63 (1.43-1.85)	<0.001	1.19 (1.04-1.38)	0.02
Nervous system disease	1.85 (1.73-1.98)	<0.001	1.47 (1.37-1.58)	<0.001
Respiratory disease	1.81 (1.68-1.96)	<0.001	1.33 (1.23-1.45)	<0.001
Chronic kidney disease	2.01 (1.85-2.18)	<0.001		
Hemoglobin (g/dL)	0.94 (0.93-0.95)	<0.001	1.02 (1.00-1.04)	0.02
White blood cell (x10^9/L)	1.06 (1.04-1.07)	<0.001	1.04 (1.03-1.05)	<0.001
Platelet (x10^9/L)	1.000 (1.000-1.000)	0.53	0.999 (0.999-1.000)	<0.001
Creatinine (µmol/L)	1.002 (1.001-1.002)	<0.001	1.001 (1.001-1.001)	<0.001
ALT (U/L)	1.001 (1.001-1.001)	<0.001	1.001 (1.000-1.001)	<0.001
Albumin (g/L)	0.94 (0.93-0.94)	<0.001	0.96 (0.95-0.96)	<0.001
Total bilirubin (µmol/L)	1.01 (1.00-1.01)	0.002		
Days from the first date of inclusion[a]	0.97 (0.96-0.97)	<0.001	1.00 (0.99-1.00)	0.01
Number of hospitalizations in the past year				
- 0	Referent		Referent	
- 1	0.53 (0.48-0.58)	<0.001	0.42 (0.38-0.47)	<0.001
- ≥2	0.88 (0.81-0.96)	0.005	0.54 (0.49-0.60)	<0.001

Use of COVID-19 oral anti-virals referred to the use of molnupiravir or nirmatrelvir/ritonavir at baseline or during follow-up. Patients were followed from the baseline date to the last follow-up date (25 April 2022), the date of first hospitalization, or the date of death, whichever came first.

[a] Days from the first date of inclusion referred to the number of days from the first date of inclusion, *i.e.,* 16 February 2022.

aHR = adjusted subdistribution hazards ratio, ALT = alanine aminotransferase, CI = confidence interval.

© 2023 Ma BHM et al. *JAMA Network Open.*

eTable 6. Univariate and Multivariable Analysis by Cox Proportional Hazard Model in the 5-Day Landmark Analysis on Factors Associated With Death/Intensive Care Unit (ICU) Admission/Use of Invasive Mechanical Ventilation (IMV) in 14,617 COVID-19 Patients Who Received Care From Community Geriatric Assessment Team

Parameters	Univariate analysis		Multivariable analysis	
	**HR (95% CI)**	***P* value**	**aHR (95% CI)**	***P* value**
Use of COVID-19 oral anti-viral				
- No oral anti-viral use	Referent		Referent	
- Use of molnupiravir	0.19 (0.16-0.23)	<0.001	0.43 (0.34-0.53)	<0.001
- Use of nirmatrelvir/ritonavir	0.04 (0.01-0.16)	<0.001	0.13 (0.03-0.52)	0.004
Age (years)	1.03 (1.02-1.03)	<0.001	1.03 (1.03-1.04)	<0.001
Male gender	1.60 (1.45-1.76)	<0.001	1.49 (1.35-1.66)	<0.001
Circulatory system disease	1.05 (0.94-1.18)	0.36	0.86 (0.76-0.96)	0.008
Digestive system disease	1.51 (1.34-1.71)	<0.001		
Diabetes mellitus	1.05 (0.96-1.16)	0.29		
Malignant tumor	1.88 (1.54-2.30)	<0.001	1.40 (1.14-1.73)	0.002
Nervous system disease	1.86 (1.66-2.08)	<0.001	1.43 (1.28-1.61)	<0.001
Respiratory disease	2.03 (1.80-2.29)	<0.001	1.40 (1.23-1.59)	<0.001
Chronic kidney disease	2.13 (1.86-2.44)	<0.001		
Hemoglobin (g/dL)	0.94 (0.91-0.97)	<0.001	1.05 (1.02-1.07)	0.002
White blood cell (x10^9/L)	1.06 (1.05-1.07)	<0.001	1.04 (1.03-1.05)	<0.001
Platelet (x10^9/L)	0.999 (0.999-1.000)	0.003	0.999 (0.998-0.999)	<0.001
Creatinine (µmol/L)	1.002 (1.002-1.002)	<0.001	1.002 (1.001-1.002)	<0.001
ALT (U/L)	1.001 (1.000-1.001)	0.001	1.001 (1.000-1.001)	0.002
Albumin (g/L)	0.91 (0.91-0.92)	<0.001	0.93 (0.92-0.94)	<0.001
Total bilirubin (µmol/L)	1.01 (1.00-1.01)	<0.001	1.00 (1.00-1.01)	0.04
Days from the first date of inclusion[a]	0.95 (0.94-0.95)	<0.001	0.97 (0.97-0.98)	<0.001
Number of hospitalizations in the past year				
- 0	Referent		Referent	
- 1	0.54 (0.46-0.64)	<0.001	0.58 (0.48-0.69)	<0.001
- ≥2	0.97 (0.84-1.12)	0.68	0.74 (0.63-0.86)	<0.001

Use of COVID-19 oral anti-virals referred to the use of molnupiravir or nirmatrelvir/ritonavir at baseline or during follow-up. Patients were followed from the baseline date to the last follow-up date (25 April 2022), the date of first intensive care unit admission, the date of first use of invasive mechanical ventilation, or the date of death, whichever came first.

[a] Days from the first date of inclusion referred to the number of days from the first date of inclusion, *i.e.,* 16 February 2022.

aHR = adjusted subdistribution hazards ratio, ALT = alanine aminotransferase, CI = confidence interval.

© 2023 Ma BHM et al. *JAMA Network Open.*

eFigure 1. Patient Flowchart

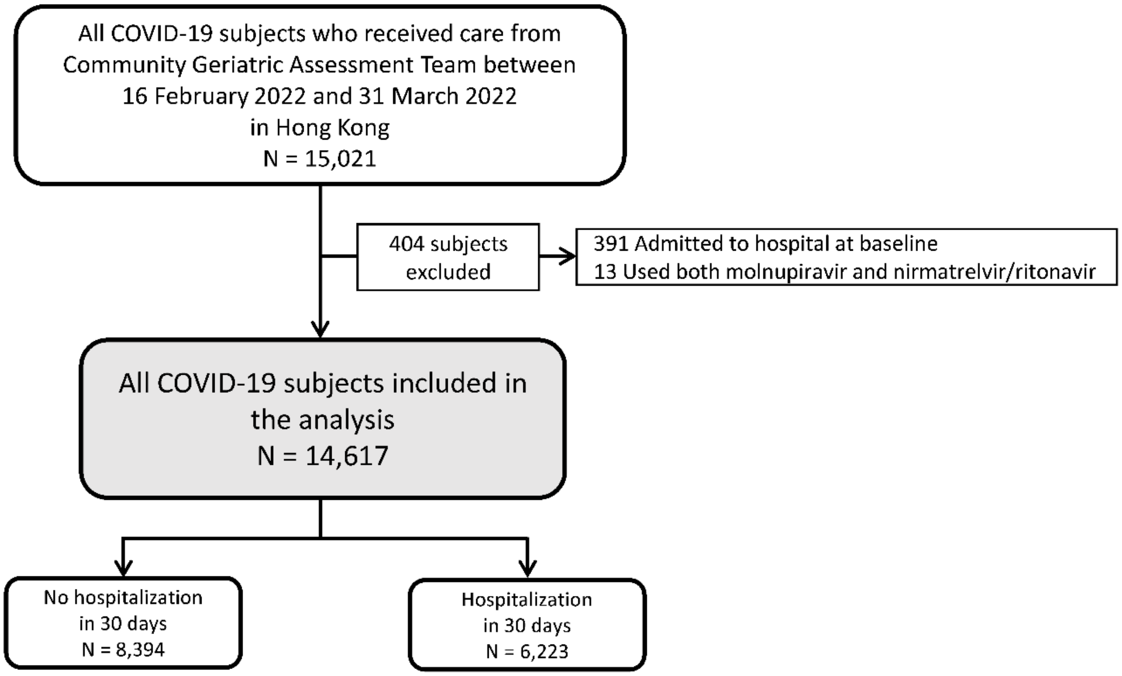

© 2023 Ma BHM et al. *JAMA Network Open.*

eFigure 2. Cumulative Incidence of (A) Hospitalization and (B) Death/Intensive Care Unit (ICU) Admission/Invasive Mechanical Ventilation (IMV) Use After Propensity Score Weighting in the 5-Day Landmark Analysis in a Single Imputed Dataset

A.

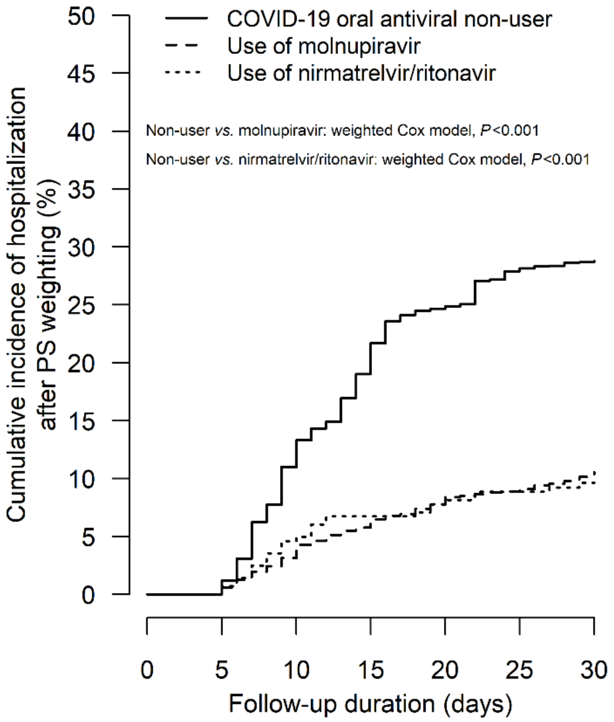

B.

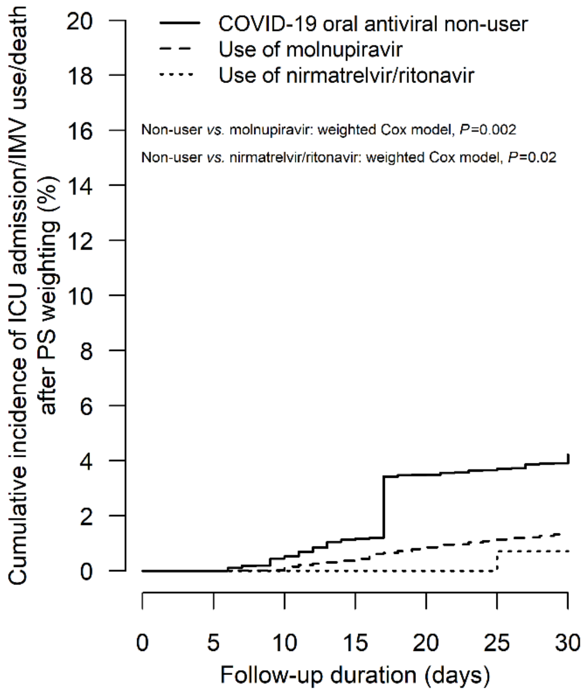

© 2023 Ma BHM et al. *JAMA Network Open.*
