# Supplementary material for: Clinical Outcomes Following Treatment for COVID-19 With Nirmatrelvir/Ritonavir and Molnupiravir Among Patients Living in Nursing Homes
Source: JAMA Netw Open. 2023 Apr 27;6(4):e2310887. doi: 10.1001/jamanetworkopen.2023.10887 (PMC10140804; doi:10.1001/jamanetworkopen.2023.10887)
Supplement: Supplement 2. — Data Sharing Statement [file jamanetwopen-e2310887-s002.pdf]

Data Sharing Statement

Ma. Clinical Outcomes Following Treatment for COVID-19 With Nirmatrelvir/Ritonavir and Molnupiravir Among Patients Living in Nursing Homes. *JAMA Netw Open*. Published April 27, 2023. doi:10.1001/jamanetworkopen.2023.10887

Data
Data available: No

Additional Information
Explanation for why data not available: All the data belong to the Hospital Authority
